# First molecular confirmation of *Coenurus cerebralis* in sheep and goats with neurological behaviors in Iraq

**DOI:** 10.14202/vetworld.2021.1420-1425

**Published:** 2021-06-03

**Authors:** Eva Aisser Ajaj, Hadeel Asim Mohammad, Hasanain A. J. Gharban

**Affiliations:** 1 Department of Internal and Preventive Medicine, College of Veterinary Medicine, University of Mosul, Nineveh, Iraq; 2 Department of Internal and Preventive Veterinary Medicine, College of Veterinary Medicine, Wasit University, Wasit, Iraq

**Keywords:** definitive, intermediate host, Iraq, polymerase chain reaction, *Taenia multiceps*

## Abstract

**Background and Aim:**

*Coenurus cerebralis* is the intermediate stage of the canine cestode, which infect sheep and goats, resulting mainly in neurological signs and causing direct and indirect economic losses. This study aimed to demonstrate the existence of *C. cerebralis* and to elucidate the role of this parasite in inducing neurological behaviors in sheep and goats.

**Materials and Methods:**

On the basis of historical data on neurological signs, we subjected 76 animals (49 sheep and 27 goats) of different ages, sexes, and geographical areas for molecular examination of their blood samples using the polymerase chain reaction assay.

**Results:**

Of the 76 animals, 23.68% tested positive for *C. cerebralis* infection. We found significant increases in infection (p<0.05) in sheep (26.53%) more than in goats (18.52%). Circling movement was prevalent significantly among both *C. cerebralis-*positive sheep and goats. The Nineveh region had a significant (p<0.05) increase in positive sheep and goats, and the sheep of all study regions were infected significantly (p<0.05) more than were the goats. We found no significant (p>0.05) variation between sheep ages ≥1-3 years and ≥3 years; however, both groups had a significantly (p≤0.043) higher positivity rate than did sheep ages <1 year. The findings of sheep ages <1 year and ≥1-3 years were significantly higher than those of the goats, but not for goats ages ≥3 years. Female sheep and goats showed a significant increase in positivity versus that for the males.

**Conclusion:**

To the best of our knowledge, this study is the first report in Iraq targeting detection of *C. cerebralis* in sheep and goats with neurological behaviors; therefore, additional studies involving different animals in other regions using molecular techniques are needed.

## Introduction

*Coenurus cerebralis* is the intermediate stage of the canine cestode *Taenia multiceps*, which mainly exists in sheep and goats but sometimes is found in cattle, camels, buffaloes, horses, monkeys, and even humans throughout much of the world, including Iraq [1,2]. The life cycle of this parasite is indirect, requiring the host to complete its development. In definitive hosts such as dogs and rarely cats and foxes, the mature cestode resides in the small intestine, and eggs are excreted daily with the feces, contaminating the environment. During grazing, intermediate hosts can be infected by ingesting the grasses contaminated with eggs. The oncospheres released from eggs penetrate the small intestine and, through blood circulation and the lymphatic system, reach most body organs and develop into a cysticercus. *C. cerebralis* may take 6-8 months to develop to its full size that appears as a single or double thin-walled, fluid-filled, cyst-like structure measuring approximately 1×1.5-4.5×7 cm in diameter [3-5].

In sheep and goats, the presence of *C. cerebralis* in the brain and spinal cord typically can lead to variable neurological signs depending on cyst location and depth. Because a long list of diseases are implicated in causing neurological signs in sheep and goats, such as listeriosis, pregnancy toxemia, middle ear infection, brain abscesses, toxicity, vitamin and mineral deficiencies, sinusitis, cerebral edema, and neoplasia [6-8], *C. cerebralis* infection (coenurosis) must be differentiated from these diseases.

Clinicopathological examinations are not used in the diagnosis of animals, and serological tests are not sufficiently specific to be of value [8]. Although postmortem findings on the cysts remain the gold standard test, molecular characterization of naturally and experimentally infected sheep and goats by polymerase chain reaction (PCR) assay shows a true positivity at 100% [9-11].

In Iraq, *C. cerebralis* is endemic in sheep and goats, resulting in direct economic losses due to morbidities and mortalities and indirect losses from low carcass weight [2,12]; however, the rate of prevalence continues to be unknown because of the absence of molecular and epidemiological studies.

This study aimed to demonstrate the existence of *C. cerebralis* and to elucidate the role of this parasite in inducing neurological behaviors in sheep and goats.

## Materials and Methods

### Ethical approval

The current study was licensed, carried out, and approved by the Scientific and Ethical Committee of the College of Veterinary Medicine, Wasit University, Wasit Province, Iraq.

### Study period and location

The study was conducted from May to September 2020 in many rural areas located in the provinces of Baghdad, Wasit, and Nineveh in Iraq.

### Sample collection

We used 76 animals (49 sheep and 27 goats) of different ages and sexes. We examined the study animals clinically to report variable neurological signs, excluding those animals exposed previously to a traumatic accident. We collected a 2.5 mL jugular venous blood sample from each animal under aseptic conditions into an EDTA anticoagulant glass gel tube. The whole-blood samples were transported to the laboratory and kept frozen at 4°C until needed for the DNA extraction.

### Sample preparation and molecular detection

Following the Type A Protocol of the G-spin Total DNA Extraction Kit (Intron Biotechnology, Korea), DNA was extracted from the whole-blood samples. The blood tubes were thawed first in a water bath at 37°C, and then, 200 μL of each blood sample was pipetted into a labeled 1.5 mL Eppendorf tube, adding 20 μL of Proteinase K, 5 μL of RNase, and 200 μL of BL Buffer. All tubes were mixed using the vortex and incubated at 56°C for 10 min. Post-centrifugation (12,000 rpm/1 min), 200 μL of absolute ethanol was added to each tube, mixed by vortex, and centrifuged (12,000 rpm/1 min), and the lysate was transported into the spin column tube. Post-centrifugation (12,000 rpm/1 min), the filtrate was discarded, and a new 2 mL collection tube was used. A total of 700 μL of WA Buffer were added to the spin column and centrifuged at 12,000 rpm for 1 min, and the flow-through was discarded; then, a total of 700 μL of WB Buffer were added, and the latter step was repeated.

After performing additional centrifugation, a total of 50 μL of CE Buffer were added to each Eppendorf tube that incubated at room temperature (25-28°C) for 2 min and centrifuged at 12,000 rpm for 1 min. To evaluate purity and concentration of the extracted DNA (DNA template), the Nanodrop system (Thermo Scientific, UK) was used.

Targeting the mitochondrial NADH dehydrogenase subunit 5 gene (*nad5*), one set of primers (nadF: 5′-GATTTAGTGGGTTTTGAGTTG-3′) and (nadR: 5′-AAAATTGCATGTAATCATAAC-3′) was used [13]. According to the manufacturer’s instructions for the Maxime PCR PreMix Kit (Intron, Korea), a tube of master mix was prepared at a final volume of 20 μL. Conditions for the thermal cycler (Bio-Rad, USA) system were designed (Table-1). Ten microliters of each PCR product in addition to 5 μL of the DNA ladder were analyzed in stained agarose gel (1.5%) with ethidium bromide. Visualization was done using the UV illuminator (Clinx Science, China).

**Table-1:** Conditions of the thermal cycler.

Cycle’s no.	Step	Temperature (°C)	Time
1	Initial denaturation	94	2 min
35	Denaturation	94	20 s
	Annealing	50	10 s
	Extension	72	20 s
1	Final extension	72	5 min
-	Hold	4	-

### Statistical analysis

We analyzed the data using the GraphPad Prism version 6.0.1.298 (GraphPad Software Inc., USA). We used the Chi-square (χ^2^) and t-tests to estimate significance at p<0.05 for positive findings for the study animals and to evaluate the association of positivity to neurological signs and epidemiological risk factors (area, age, and sex), respectively.

## Results

**Figure-1:**
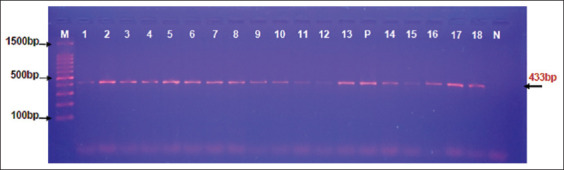
Agarose gel electrophoresis of positive DNA samples to *C. cerebralis* at 433 bp. Lane (M): Ladder marker (1500-100 bp); P: Positive control; N: Negative control. Lanes 1-13: Positive sheep; Lanes 14-18: Positive goats.

The collected case history data and clinical examination of the study animals, before the PCR estimation of the blood samples, showed a significant variation (p<0.05) in their values. In addition, some clinically evaluated animals showed >1 abnormal sign. However, higher significant (p<0.05) values detected in sheep were circling movement (94.44%) and retardation from the flock (86.21%); in goats, these included depression (87.5%) and convulsion (85.71%). The sheep also showed a significant increase (p<0.05) in head shaking (77.42%), head deviation (75%), uncontrolled movement (77.78%), and blindness (66.67%) (Table-2). Of the 76 study animals tested using the conventional PCR assay, 18 (23.68%) were positive for *C. cerebralis*. We found significant increases (p≤0.037) in the positivity rate in the sheep (13/49 [26.53%]) versus the goats (5/27 [18.52%]) (Table-3 and Figure-1).

**Table-2:** Neurobehavioral signs of study animals before polymerase chain reaction performance, n=76.

Neurological sign	Total no.	Sheep	Goats	*χ^2^*	Significance
Circling movement	36	34 (94.44%)*	2 (5.56%)	11.331	S
Head shaking	31	24 (77.42%)	7 (22.58%)	9.485	S
Retardation from flock	29	25 (86.21%)*	4 (13.79%)	10.122	S
Depression	16	2 (12.5%)	14 (87.5%)*	9.918	S
Head deviation	12	9 (75%)	3 (25%)	8.34	S
Uncontrolling movement	9	7 (77.78%)	2 (22.22%)	9.624	S
Convulsion	7	1 (14.29%)	6 (85.71%)*	10.007	S
Blindness	3	2 (66.67%)	1 (33.33%)	7.319	S
p-value	0.036	0.011	

* Significance (p<0.05), S=Significant

**Table-3:** Positive results of polymerase chain reaction assay in study animals, n=76.

Animal	Total no.	Positives (%)	p-value
Sheep	49	13 (26.53)*	0.047
Goats	27	5 (18.52)	
Total	76	18 (23.68)	

* Significance (p<0.05)

We saw significant variation (p≤0.048) in the distribution of the findings of positivity (Table-4). The findings of depression (56.25%) and head shaking (19.35%) showed a significant increase and decrease, respectively, among the animals positively infected with *C. cerebralis*. Findings of blindness, head deviation, circling movement, retardation from the flock, and uncontrolled movements were 42.86%, 41.67%, 38.89%, 37.93%, and 33.33%, respectively. However, we did not detect convulsion in *C. cerebralis-*positive animals.

**Table-4:** Distribution of polymerase chain reaction positive to the nervous signs, n=18.

Sign	Positive/tested	Prevalence (%)	p-value
Circling movement	14/36	38.89	0.048
Head shaking	6/31	19.35	
Retardation from flock	11/29	37.93	
Depression	9/16	56.25*	
Head deviation	5/12	41.67	
Uncontrolling movement	3/9	33.33	
Blindness	3/7	42.86	
Convulsion	0/3	0	

* Significance (p<0.05)

In the positive sheep, we detected a higher significance in circling movement (85.71%), while finding a significant decrease in depression (33.33%) and head deviation (40%) (p≤0.027). The prevalence of retardation from the flock, blindness, and head shaking was 72.73%, 66.67%, and 50%, respectively (Table-5). In the positive goats, depression (66.67%), uncontrolled movement (66.67%), and head deviation (60%) increased significantly (p<0.035), while circling movement (14.29%) had lower values. For head shacking, blindness, and retardation from the flock, the findings were 50%, 33.33%, and 27.27%, respectively (Table-6).

**Table-5:** Distribution of nervous signs in relation to polymerase chain reaction positive in the sheep, n=13.

Sign	Positive sheep/total	Prevalence (%)	p-value
Circling movement	12/14	85.71*	0.035
Head shaking	3/6	50	
Tendency to keep away from flock	8/11	72.73	
Depression	3/9	33.33	
Lateral deviation of head	2/5	40	
Incoordination and ataxia	1/3	33.33	
Unilateral blindness	2/3	66.67	

* Significance (p<0.05)

**Table-6:** Distribution of nervous signs in relation to polymerase chain reaction positive in the goats, n=5.

Sign	Positive sheep/total	Prevalence (%)	p-value
Circling movement	2/14	12.29	0.026
Head shaking	3/6	50	
Tendency to keep away from flock	3/11	27.27	
Depression	6/9	66.67*	
Lateral deviation of head	3/5	60	
Incoordination and ataxia	2/3	66.67*	
Unilateral blindness	1/3	33.33	

* Significance (p<0.05)

**Figure-2:**
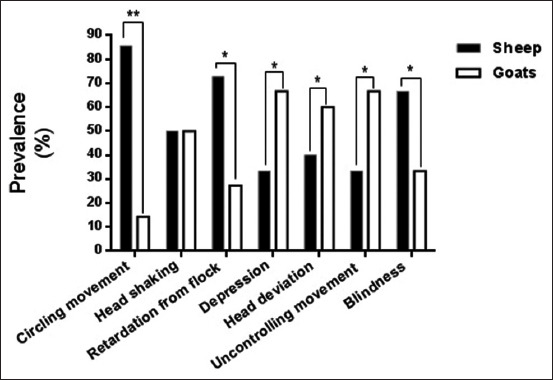
Comparative distribution of nervous signs in polymerase chain reaction (PCR)-positive sheep and goats. The positive infection of *Coenurus cerebralis* in sheep and goats was investigated by conventional PCR. The positive results of both sheep and goats were distributed to the nervous signs and the comparison between them in respect to nervous signs was performed. The data were analyzed by GraphPad Prism using t-test.

Comparing the sheep and goats, the findings of neurological signs showed a significant variation (p≤0.047) in their values (Figure-2). In sheep, circling movement, retardation from the flock, and blindness increased significantly, whereas in goats, depression, uncontrolled movement, and head deviation increased significantly. However, we found no significant ­differences (p>0.05) for head shaking (Figure-2).

For demographic risk factors, the findings showed a significant variation in their values (Table-7). For region, we detected significant increases (p<0.05) in *C. cerebralis-*positive sheep and goats of the Nineveh Province, 36.36% and 25%, respectively, while we detected significant decreases in the sheep and goats of the Baghdad Province, 18.75% and 0%, respectively. Comparatively, the findings of positive sheep among all study regions increased significantly more than that for the goats (p<0.05). Although we found no significant (p>0.05) differences between the positive sheep ages ≥1-3 years (28.57%) and ≥3 years (29.63%), findings of both groups were higher than those reported for sheep ages <1 year (12.5%), (p≤0.043). In goats, we saw significant increases (p≤0.024) in positivity in goats ages ≥3 years (33.33%) in comparison with those ages ≥1-3 years (16.67%) and <1 year (0%). Females sheep (27.91%) and goats (20%) showed a significant elevation (p<0.05) in ­positivity when compared to the males, 16.67% and 0%, respectively.

**Table-7:** Distribution of polymerase chain reaction positive among demographic risk factors (age and sex).

Factor	Sheep		Goat		*χ^2^*	Significance
	
	Positive/tested	Prevalence (%)	Positive/tested	Prevalence (%)		
Region (Province)						
Baghdad	3/16	18.75	0/5	0	5.973	S
Wasit	6/22	27.27	3/18	16.67	5.328	S
Nineveh	4/11	36.36*	1/4	25*	4.119	S
p-value		0.031		0.027		
Age (year)						
<1	1/8	1 (12.5%)	0/3	0	4.027	S
≥1-3	4/14	4 (28.57%)*	3/18	16.67	5.646	S
≥3	8/27	8 (29.63%)*	2/6	33.33*	2.05	NS
p-value		0.043		0.024		
Sex						
Female	12/43	27.91	5/25	20	3.926	S
Male	1/6	16.67	0/2	0	4.601	S
p-value		0.045		0.017		

* Significance (p<0.05)

## Discussion

In livestock production, parasitic infections continue to cause great economic losses. *C. cerebralis* is common in almost every part of the world, but it is more prevalent in underdeveloped countries [14]. In the present study, clinical screening of small ruminants undergoing neurobehavioral signs revealed that sheep cases are more available than are goat cases. Sonmez *et al*. [15] suggested that differences in clinical and pathological appearances may be explained by a genetic intraspecific variability within the species.

Parihar [16] and Gicik *et al*. [17] have shown that *C. cerebralis* can be localized to any part of the brain, mostly in the cerebral hemispheres; other studies have detected that *Coenurus* localizes mainly in the brain cortex [14] and cerebellum [15]. Variable neurobehavioral signs may be caused by extensive tissue damage, including traumatic destruction. However, the size and location of the parasite appear to be important in pathogenesis. The predilection site of the *Coenurus* cyst in most cases is the central nervous system and spinal cord [18].

This study found that the total infection rate was 23.68% in Iraq. This result is apparently similar to that obtained in Iraq [2], Turkey [19], and Iran [20], lower than recorded in Ethiopia [21] and Egypt [22], and higher than carried out in India [23] and Jordan [24]. This variation in the prevalence of disease may reflect the different management systems, the amount of contamination of the pastures with the tapeworm eggs, and the attempts for controlling and preventing infection [25,26]. In addition, the methods of selecting the samples and the techniques applied to detect infection could probably have effective roles in determining prevalence [27].

Significantly, our findings show that sheep are more sensitive to *C. cerebralis* than are goats, which is compatible with the findings of other researchers [1,17]. Low prevalence of *C. cerebralis* in goats may be attributed to fact that the primary site of infection in the goats is the muscles of the higher shoulder, especially the biceps femoris and triceps, followed by the abdominal muscles, but not in the brain and spinal cord [1,28]. We detected a significant increase in circling movement among the positively infected sheep that might be attributed to the fact that the brain is the predilection site of *Coenurus* cyst and that the compulsive circling behavior commonly observed is due to the extensive effects of chronic coenurosis, which can lead not only to more cavitation in the cranium but also to perforation and atrophy of the cranial bones [7,29]. Scott [29] has mentioned that the narrow diameter circles suggest involvement of the basal nuclei at a deep location within the forebrain, whereas wide circles suggest a more superficial location for the cerebral cyst.

Studies to determine the prevalence of *C. cerebralis* in sheep and goats have shown highly variable results. This variation could be attributed to numerous factors, including ecological diversity, geographic location, and sociology and economics, all of which can play an important role in the epidemiology of coenurosis. For the hosts, factors can include the feeding habits of carnivores, the characteristics of the slaughter process, the practice of deworming, the presence and abundance of dogs or other intermediate hosts, the type of husbandry, and the nature of the production system [30,31].

Differences in the prevalence of *C. cerebralis* among study regions could be related to the management system, the therapeutic or prophylactic veterinary intervention of the definitive host, herd ­immunity or host resistance, the density of infective stage, body condition, or a combination of these factors. The finding of a significant increase in positive *Coenurus* infection for both sheep and goats older than 1 year of age is in contrast to findings reported recently [15] and previously [32] of *C. cerebralis* affecting sheep during their 1^st^ year, mainly because small lambs of 3-4 months are left in the grass at the beginning of the spring season due to their still-developing immune system and rumen activity. However, the findings of our study were similar to those detected by Gicik *et al*. [17] that sheep age 1-2 years (15% and 21.7%, respectively) were more susceptible to the parasite, perhaps because of inadequate acquired immunity.

Oryan *et al*. [1] have reported that sheep and goats of all ages are susceptible to coenurosis, presenting a serious economic impact for the owners of these animals. Furthermore, in our investigation, most of the positive animals showing neurobehavioral signs were female, particularly the goats. These discrepancies could be attributable either to the genetic variability between males and females or to management factors.

## Conclusion

This is the first study to examine the molecular correlation of *C. cerebralis* to neurobehavioral signs in sheep and goats. Based on the findings, we conclude that molecular techniques can serve reliably to diagnose infection in the blood. In addition, many neurological signs were attributed, erroneously during clinical examination to coenurosis. Hence, neurobehavioral etiologies should be clarified depending on the available molecular and genetic tools. Furthermore, intraspecific variations within *C. cerebralis* may have biological and epidemiological significance that warrants further studies. The current study is interesting as early molecular diagnosis of *Coenurus* infections in sheep and goats and can help greatly in the successful treatment of these cases, and then decreasing direct and indirect economic losses.

## Authors’ Contributions

EAA and HAM: Collection of blood samples and molecular examination. HAJG: Clinical examination of study animals, molecular examination, and statistical analysis of study results. All authors read and approved the final manuscript.
